# What Is Wrong with Empirical-Legal Research into Victimhood? A Critical Analysis of the Ordered Apology and the Victim Impact Statement

**DOI:** 10.1093/ojls/gqaa048

**Published:** 2020-11-24

**Authors:** Vincent Geeraets, Wouter Veraart

**Affiliations:** 1 Assistant professor, Department of Legal Theory and Legal History, Vrije Universiteit Amsterdam; 2 Professor of Legal Philosophy, Department of Legal Theory and Legal History, Vrije Universiteit Amsterdam. Email: w.j.veraart@vu.nl

**Keywords:** apologies, victim impact statement, victimology, procedural justice, legal method

## Abstract

The central question in this article is whether an empirical-legal approach to victimhood and victim rights could offer a sufficient basis for proposals for reform of the legal system. In this article, we choose a normative-critical approach and raise some objections to the way in which part of such research is currently taking place, on the basis of two examples of research in this field, one dealing with compelled apologies as a remedy within civil law and the other with the victim impact statement within criminal law. In both cases, we argue, the strong focus on the measurable needs of victims can lead to a relatively instrumental view of the legal system. The legal system must then increasingly be tailored to the wishes and needs of victims. Within this legal-empirical, victim-oriented approach, there is little regard for the general normative principles of liberal democratic legal systems, in which an equal and respectful treatment of each human being as a free and responsible legal subject is a central value. We argue that results of empirical-legal research should not too easily or too quickly be translated into proposals for legal reform, but first become part of a hermeneutical discussion about norms and legal principles, specific to the normative character of law and legal science.

## 1. Introduction

Victims have become increasingly important within the context of legal proceedings. The most noticeable development in this respect is the introduction and enhancement of the victim impact statement, which is now part and parcel of many jurisdictions in the Western world. Alongside these societal and legal developments, scientific interest in victims has grown considerably and has given impetus to a relatively new field of inquiry: victimology. Our interest in this article is in victimological research with the following two core characteristics: (i) the orientation is *evidence-based*, ie surveys and interviews are at the heart of the investigation. These tools are employed in order to determine how the encounter with the law affects the victim and in particular whether it alleviates suffering or perhaps results in secondary victimisation; and (ii) this orientation is combined with an *activist* agenda. A central claim within victimology literature is that the victim has long been a forgotten party within criminal law and that this should be amended by transforming the legal arena in order to strengthen the victim’s position.^1^ The call for victim emancipation within criminal legal proceedings is the driving force fuelling part of the research. So, our concern lies with victimological research in which empirical insights are at the basis of proposals for reforming the penal legal system.^2^

We are sceptical about the fruitfulness of a part of this empirical-activist body of research. Our main critique is that within this research—due to its empirical orientation—the normative discussion often remains underdeveloped. In this regard, we identify two related problems. First, while it is common practice within victimological research to investigate how a certain reform proposal relates to law, often the inquiry does not amount to more than identifying some legal concerns and suggesting possible solutions. What is lacking is a principled discussion in which central values of the legal system itself are drawn in to find out whether the proposal can withstand scrutiny in terms of coherence and justice. Consequently, the claim of the victim being a forgotten party and the wish for reform of the law are far more problematic than victimologists commonly assume. The result of providing the victim with more and more procedural rights is that it becomes increasingly difficult to adhere to principles and values generally regarded as central to a just legal system. Secondly, victimologists insufficiently recognise the schism between empirical research and normative evaluation. It is worth repeating that normative concepts cannot be understood or established merely empirically. The concepts of *humiliation* (section 2) and *procedural justice* (section 3) will be analysed to underscore this point.

In order to cement our critique, we discuss two examples of recent empirical-legal research into victimhood. The first example concerns the *ordered apology*, which we discuss in section 2, and the second example is the *victim impact statement*, which we investigate in section 3. These examples were by no means randomly chosen. Apology to the victim is an important topic within empirical-legal research in the context of civil law, while the same holds true in relation to the victim impact statement, but in the context of criminal law. In both examples, we focus on publications that carry a certain gravitas and can therefore be expected to be indicative of a more general problem. Importantly, in this article, it is not our goal to question the validity or reliability of the empirical data and insights generated by evidence-based research itself. That is a task reserved for scientists who are well versed in carrying out this kind of research. Rather, we simply assume that the empirical insights in question are well founded and start our discussion from there. In section 4, we summarise our conclusions and sketch the outlines of what a more critical, value-based approach into the legal accommodation of victim needs could consist of. Though our approach does not deny either the fact that victim needs exist in many forms or that they ought to be taken care of, it is sceptical of the use of legal institutions as a *direct* way of recognising victims.

## 2. Ordered Apology

Victimologists indicate that, in terms of recovery, the emotional needs of the victim may be more important than material needs.^3^ In a recent article in OJLS, Gijs van Dijck picks up on this line of thinking.^4^ He investigates whether the *ordered apology* could be a useful instrument to serve some of these emotional needs. The main idea is that victims should not only be able to sue for compensation, but also be allowed to claim an apology (as a remedy). Central to Van Dijck’s analysis are insights derived from psychological studies. Our main concern in this section is with his analysis. We will also critically assess related publications by Andrea Zwart-Hink, Arno Akkermans and Kiliaan van Wees, Aaron Lazare and Brent White.^5^

Van Dijck starts out his discussion by indicating what it means to apologise and what kind of requirements should be fulfilled before recipients can accept an apology. After this general introduction, he addresses an important issue: can ordered apologies be considered beneficial or is the fulfilment of such an obligation likely to be insincere and therefore without value? In our estimation, the prevailing view is that compelling someone to apologise is highly problematic, because such an apology is not the authentic expression of that person.^6^ It cannot be regarded as indicative of a sense of guilt, regret or remorse on her part about the harm she caused and therefore provides an unsuitable basis for a (partially inward) process of atonement and reconciliation. Interestingly, however, Van Dijck reaches a different, counter-intuitive conclusion. He states that:

Even though sincerity is important, the sincerity argument is flawed. First, seemingly voluntary apologies may not be truly authentic either, or they may not come across as authentic. Secondly, while it may be true that ordered apologies lack sincerity, it is not necessarily true that such apologies have no utility. It turns out that sincerity is not the sole predictor for the apology being accepted by the victim.^7^

However, the first part of this argument is a *non sequitur*. The fact that non-coerced apologies may not always be authentic either cannot be considered a sufficient response to the moral objection that coercion precludes authenticity and that therefore it is wrong to compel an adult to apologise.^8^ From a moral point of view, it matters whether authenticity is possible at all. That the sincerity argument is flawed can thus only be established by means of the second part of the argument, which stresses the possible utility of the ordered apology. Van Dijck fleshes out this argument by referring to several psychological studies. These studies reveal that although people do prefer a sincere apology, they also appreciate receiving an apology even when they have strong reasons for suspecting that it was not made sincerely.^9^ Victims can benefit from an ordered apology, according to Van Dijck, as it may mitigate stress, restore their social standing and facilitate healing by offering acknowledgement, validation and/or closure. Claimants may experience the receiving of an apology as a vindication, which, in turn, may restore their dignity and self-respect. An ordered apology may also be beneficial to society in general as it could serve as a deterrent by drawing public attention to the fact that this kind of behaviour is considered wrong.^10^

Van Dijck qualifies his position in a number of ways. First, he stresses that a judge should sometimes deny a claim for apology. To this end, he proposes a proportionality test to assess whether ordering an apology is appropriate. A relevant factor according to Van Dijck is the amount of injustice involved. If the injustice is relatively small, it could be disproportionate to require the defendant to apologise. Secondly, the severity of the sanction may also be mitigated in other ways. For example, the judge may require the defendant to offer the apology only in private, or in public without much specificity. Finally, a judge could order the apology without providing a sanction in the case of non-compliance. These possibilities to alleviate the sanction, Van Dijck indicates, are important in relation to the human rights of the defendant. *Prima facie*, an order to apologise violates one’s freedom of expression as one is forced to make a statement with which you may not agree. However, according to Van Dijck, the ordered apology—especially when the above-mentioned mitigations are in play—need not amount to a violation, because this freedom—as established in the European Convention on Human Rights (ECHR)—is not an absolute right. Van Dijck shows that the European Court of Human Rights has in fact validated the ordered apology in a certain number of cases.^11^

Van Dijck, and also Zwart-Hink, Akkermans and Van Wees, primarily appeal to psychological studies to support their claim that ordered apologies have utility. Victims value an apology even when the other party apologises only because an authority has ordered her to do so. From a legal perspective, these are *prima facie* interesting facts, but they are not of decisive normative importance. In general, the fact that people value X does not necessarily mean that the introduction of X in the legal system will be a good thing. Drawing such a conclusion would be fallacious in light of the fact–value distinction. Suppose, for example, that a certain class of victims turn out to desire the mutilation of the person who has injured them. Rather than legally implementing this desire, it should be conveyed to them that such a wish can never be fulfilled in a civilised system of law. To quote Michael Moore: ‘we ought to educate citizens to the right view, not reinforce them in their error’.^12^ Personal needs or preferences are relative to time and place, and are on occasion incompatible with central values of the legal system. The fact that people value a certain measure or arrangement may be a starting point for reflection or debate, but it is unacceptable as the final line of reasoning in an argument which purports to reshape the law.

From a methodological point of view, it is desirable that critical questions are asked about the ordered apology and how this instrument interacts with central values of the legal system. An important preliminary question is: why do victims value an apology even if they know that it is not the authentic expression of the defendant? Strangely enough, this preliminary question receives very little attention in both articles. Zwart-Hink, Akkermans and Van Wees do not even attempt to address it, whereas Van Dijck only mentions in passing that the victim’s feeling of vindication may be caused by the humiliation of the defendant.^13^ Aaron Lazare, an author approvingly referred to in both articles, is more explicit. According to Lazare, humiliation is an important victim’s motive for ordering the apology.

Lazare elucidates this claim, among others, by means of a historical example, dating from the Second World War. When visiting a hospital ward during the military campaign of the allied forces in Sicily 1943, the American general George Patton slapped two soldiers under his command, because he believed them to be feigning illness. The incident led to a public scandal after Patton had been bragging about his behaviour. The supreme commander of the Allied forces, Dwight Eisenhower, was faced with a dilemma. On the one hand, it was very important to retain his most effective general in the field, but on the other hand, this kind of behaviour could not be tolerated. Eisenhower solved the thorny issue by ordering Patton to publicly apologise for what he did. In this context, Lazare states that:

His alternative to dismissing Patton was to *humiliate* him by demanding an apology … Apologies, genuine or not, can be effective (or regarded as successful) when the offender is humiliated and the offended has their dignity restored. This was the case with the Patton apology. Respect for medical patients and medical facilities was restored by Patton’s apologies. Even more important, the other offended parties—reporters, legislators, and the American public—took satisfaction in seeing Patton *punished*.^14^

The fact that Van Dijck and Zwart-Hink, Akkermans and Van Wees do not discuss the theme of humiliation leads us to believe that they are, unjustly, not genuinely interested in this topic. However, this observation also holds good in relation to Lazare. He indicates that Eisenhower’s dilemma was effectively solved by means of the ordered apology, but he neglects to mention that historical reality was in fact much more complicated. Patton reluctantly apologised in private to the soldiers in question, but when he was about to make public apologies in front of 3000 soldiers, the crowd prevented him from speaking by kicking up an infernal racket. The soldiers recognised the humiliating element in the ordered apology and protested against it in response.^15^

The theme of humiliation raises a host of questions. A relevant question in the present context is: how does a humiliating measure relate to the central values of the legal system? This question pertains not only to the freedom of expression, but also to the value of human dignity^16^ and the prohibition on inhuman or degrading treatment.^17^ Furthermore, the introduction of the ordered apology raises issues of coherence within the legal system itself. For example, should performing the order be regarded as an apology under duress? If so, how does this relate to a statement made under duress which renders a contract void? As long as these normative issues are not addressed, it is hard to see how any sound recommendations can be made about the introduction of the ordered apology in the legal system. We, therefore, take issue with the position of Zwart-Hink, Akkermans and Van Wees as they encourage victims to claim ordered apologies without considering how the awarding of such a claim relates to the dignity and rights of the defendant.^18^ Van Dijck, in contrast, is more cautious as he explicitly restricts the scope of his own contribution at the start of his discussion. He states that although it is an assumption of the article ‘that the law should address or recognise victims’ desire for an apology’, his goal merely is to provide correct information in order to facilitate a subsequent normative debate. He indicates that any political discussion about whether it is desirable to introduce the ordered apology within civil legal proceedings falls outside the scope of his article.^19^ The implication seems to be that he need not address these questions.

This proviso is, of course, legitimate. However, as it turns out, Van Dijck’s article does much more than just providing the reader with factual information. It is in fact very much concerned with normative issues and assessing the legal system. Consider, for example, Van Dijck’s (already discussed) idea of proportionality in light of a possible infringement of the freedom of expression. As indicated, Van Dijck maintains that ordering an apology could, *under certain circumstances*, be disproportionate as a sanction. This way of looking at things, however, neglects the legal possibility that an ordered apology should *always* be considered excessive because of the inherent humiliating element involved.^20^ Also, Van Dijck continually expresses himself in terms of utility and effectiveness, and proposes numerous arguments in favour of the legal introduction of the ordered apology. In contrast, the tension between humiliating the defendant and respecting her as a responsible legal subject does not come into view: it appears to be a blind spot in Van Dijck’s take on the issue.

Van Dijck’s proportionality proposal may have been influenced by an earlier, related article of Brent White.^21^ Similar to Van Dijck’s article, White adopts an instrumental approach to the law, with a strong focus on the utility and effectiveness of measures. Just like Van Dijck, White considers the sincerity argument flawed because of the possible utility of the ordered apology. In contrast to Van Dijck, White regularly mentions the humiliating element of the ordered apology, but, similar to Van Dijck, he does not consider the possibility that it can give rise to a principled normative objection. Throughout the article, White adopts economic metaphors in his discussion to emphasise the appropriateness of ordering a defendant to apologise. He noteworthily characterises the ordered apology as ‘a symbolic *transfer* of humiliation and power between the offender and the victim’.^22^ Humiliation in White’s analysis is presented as a commodity, which can be exchanged and traded off. He calls this an ‘*exchange* of humiliation’.^23^ The basic idea appears to be that if by doing wrong a defendant humiliated the victim, a judge may in response humiliate the defendant by ordering her to apologise. This, in turn, connects well with Van Dijck’s proportionality proposal: increasingly serious wrongdoing merits increasingly serious humiliation. However, apart from the appropriateness of the exchange metaphor used by White, can this assumption of humiliation meriting further humiliation withstand scrutiny in light of the central values of the legal systems involved?

There exists an elaborate legal-philosophical debate on the ordered apology in relation to humiliation which neither of these authors addresses. Immanuel Kant, noticeably, considered an ordered apology to be justified as punishment in the context of criminal law, if humiliating the offender is required in light of *ius talionis*.^24^ However, it is questionable whether Kant’s view on the matter is consistent with his more general views on deontological ethics. Central to Kant’s ethical thinking is the idea that a person should also always be respected as an autonomous agent, which appears to include that she may not be deprived of the freedom of her own voice. Nick Smith concurs with this line of thought. He maintains that in criminal and civil law, offenders should never be ordered to apologise, because this theatrical form of humiliation alienates them from both the community and themselves.^25^ In the same vein, Linda Radzik states that ‘the history of atonement is in large part a history of degradation’.^26^ Considered from a modern legal-historical perspective, the ordered apology appears to come close to the compelled public confession. This tool, notoriously utilised during the Soviet era but also currently in vogue in China, exemplifies legal systems in which individual rights are completely subordinate to what is perceived as the common good.^27^

Avishai Margalit provides an interesting analysis of the concept of humiliation in his celebrated work *The Decent Society*. He maintains that only a society ‘whose institutions do not humiliate people’ can be considered decent.^28^ A central element in humiliation is rejection of the person from what Margalit calls the ‘family of man’. Rejection takes place when institutions do not recognise individuals as responsible agents with a dignity of their own, but treat them as if they are objects, animals or children.^29^ Humiliation through rejection, Margalit argues, is detrimental to self-respect as individuals lose control over their lives by not being recognised as responsible agents. Following this analysis, it is not difficult to recognise humiliating features in the ordered apology. By forcing adults to apologise for their actions, they are treated as if they were children who, typically, are urged on by their parents or teachers to say sorry to classmates for what they did. Rendering adults infantile—treating them as if they are children—is a well-known humiliating practice^30^ rooted in, among other practices, (colonial) racism and slavery.^31^ The loss of one’s voice—the individual cannot control what she is forced to say—provides an indication of a violation of the person’s dignity as one is alienated from one’s own statements.^32^ However, Van Dijck has no regard for this problem when he states: ‘Parents make their children apologise. Why would the legal community not follow the parents’ pedagogic example?’^33^

By being overly focused on quantifiable goals such as utility and effectiveness, Van Dijck, Zwart-Hink, Akkermans and Van Wees, Lazare and White fail to appreciate that the legal system also represents values of a different, non-quantifiable character, such as human dignity. As Margalit pointedly states: ‘If there is no concept of human dignity, then there is no concept of humiliation either.’^34^ Importantly, human dignity and humiliation are normative rather than psychological concepts, deriving their legal meaning primarily from their (positive or negative) connection to central legal values. It would be a serious mistake to presuppose that what counts as humiliation can only or sufficiently be understood empirically by mapping out when people actually feel humiliated.^35^ Margalit’s observation that humiliation is primarily a normative concept is in line with our objection to the approach in the publications we have previously discussed. In their strong focus on the measurable needs of victims, the authors embrace a predominantly instrumental approach to positive law, with the result that as fundamental a normative (non-utilitarian) legal principle as the respect for the human being as a responsible person remains largely outside their view. Partly because of this, they do not seem to be able to critically assess the—also primarily normative—humiliating element in the compelled apologies and discuss the possible legal implications thereof in their line of argument.

In our view, these publications fall short in methodological terms. What they lack is a value-based approach, in which jurisprudence is primarily understood as an autonomous science with its own hermeneutical method of interpreting, arguing and valuing—more closely related to the humanities than to the social sciences. Although legal science can be strengthened and enriched with observations and insights from other disciplines such as psychology and other social sciences, a normative reflection on and evaluation of these insights in light of basic normative principles that underlie the legal system as a whole is always needed. Therefore, an empirical-legal scholar should not only discuss how the empirical data yielded from the other discipline should influence the way in which the positive law currently functions and is applied, but also how these empirical data, and the legal reform they appear to promote, relate to central values and principles of the legal system involved. The researchers must clarify their own position with regard to these basic values; of course, there are several possibilities to understand and interpret the normative framework on which the positive law is based.^36^

## 3. Victim Impact Statement

Our second example concerns the victim impact statement (VIS), which has been introduced in many jurisdictions in the Western world. Our interest lies in a critical discussion of some arguments used to promote VIS, not in its precise—and sometimes tense—implementation within criminal procedural law in different legal systems. While VIS has received a critical reception within legal and philosophical literature,^37^ victimologists tend to welcome it as one of the most tangible results of victim emancipation.^38^ According to victimologists, VIS enhances the fairness of the trial by providing the victim with voice. At first sight, their discussion appears to be value-based, since normative concepts such as *voice* and *procedural justice* figure prominently in these studies. However, on closer inspection, it emerges that these concepts are almost exclusively based on empirical sources, by means of surveys and interviews. Our main line of argument in this section is that, in particular, the empirical understanding of the concept ‘procedural justice’ is problematic from a methodological point of view. In addition, just as in the previous section, we argue that empirical findings cannot directly support a call for legal change, but, rather, should first become part of a hermeneutical discussion about the legal values and principles specific to the normative character of positive law and legal science. In our analysis, we focus on various publications by Edna Erez and Jo-Anne Wemmers. We also discuss the work on procedural justice by Tom Tyler and Allan Lind.

While victimologists advance many arguments in favour of VIS, the arguments related to procedural justice and therapeutic jurisprudence seem most significant. In the first argument, it is advanced that the implementation of VIS contributes to the fairness of legal proceedings. VIS enables victims to actively engage in legal proceedings and this provides them with what is called ‘voice’. Both Erez and Wemmers equate voice with the notion of ‘process control’.^39^ Their line of argument is that providing room for the victim’s voice enhances their sense of process control, which, in turn, strengthens the value of procedural justice. The second argument is based on the assumption that VIS should be welcomed from a therapeutic point of view.^40^ According to Erez and Wemmers, being allowed to make a statement in court can positively influence the victim’s mental condition and well-being, while the feeling of being silenced is detrimental to recovery. Both scientists address the collateral problem of secondary victimisation. In order to minimise this danger, it is important, they indicate, to explain to victims in advance what they might experience in the courtroom. Moreover, Erez argues that judges should explicitly acknowledge the input of victims, for example, by using phrases from their impact statement in sentencing comments. Victims would appreciate this as a source of satisfaction.^41^

*Prima facie*, the argument relating to procedural justice seems the more fundamental of the two as it represents a value which is firmly entrenched within the legal system itself. Or, to put it differently: suppose, hypothetically speaking, that VIS would have a negative impact on the fairness of proceedings. In that case, it does not seem sensible—notwithstanding possible therapeutic effects—to address the needs of victims by means of VIS, which implies that the argument of procedural justice takes priority. But can this argument based on procedural justice withstand scrutiny? Do legal proceedings become more just by the inclusion of VIS? Crucially, Erez and Wemmers (and victimologists in general) rely on *victim perceptions* of procedural justice in answering these questions. Erez reports that victims value VIS as it enhances their satisfactions with justice and adds to their sense of fairness.^42^ Wemmers, meanwhile, analyses the concept of procedural justice by investigating how victims assess the fairness of legal proceedings and how this correlates with their judgments concerning the friendliness of the judge, the interest and consideration shown, the opportunity to express one’s views, the honesty of the judge, the absence of bias and the accuracy of the decision.^43^ A major influence on this empirical way of viewing the matter is the work of Tyler and Lind.^44^ It is illuminating to discuss the analysis of these famous predecessors and relate it to the argument of procedural justice as advanced by Erez and Wemmers.

Under what conditions can authority function effectively? Tyler and Lind indicate that *legitimacy* is a very important factor as it is almost always crucial for an effective exercise of authority. People comply with decisions because they consider their source legitimate. This judgment of legitimacy, in turn, depends in large part on *how* the decision maker has reached her verdict. If people consider the decision process to be fair, the authority is acknowledged, and *vice versa*. So, perceptions of procedural justice determine to a large extent whether the decision maker is considered legitimate. But why is that? Why do people consider the fairness of the procedure so important? A first explanation is that the procedural fairness serves some external goal(s). Tyler and Lind call this an *instrumental* model. For example, parties may consider procedural fairness important because it ensures consistency in the decisions, which, in turn, enables parties to cooperate efficiently. Cooperation is the external goal in the example. At first glance, the instrumental model appears attractive. It offers, or so it seems, an explanation for why parties value being enabled to present information to the decision maker: they can maintain a feeling of control and hope for a decision that favours them or, at least, takes their arguments into consideration.^45^

However, by means of experiments, Tyler and Lind reveal some inadequacies of the instrumental model. In one of their experiments, participants were informed that the decision maker was interested in hearing what they had to say, but that their contribution would not change the goal that was already set. Still, parties considered the opportunity to make a contribution as adding significantly to the fairness of the procedure.^46^ The study indicates that the outcome of the decision may matter less to victims than the feeling of being heard *in and of itself*.^47^ In this context, as an alternative to the instrumental model, Tyler and Lind formulate a *relational* model. The gist of this model is that people value a positive relationship with authority—for example, the decision maker listens attentively to what they have to say—because it offers an indication that they are valued members of society, whereas a negative relationship implies that they have a low-status position. If the relationship is positive, the procedure is considered fair and the authority legitimate, and *vice versa*. The factors ‘standing’, ‘neutrality’ and ‘trust’ turn out to be especially important in the perception of procedural justice. These factors can all be understood in relational terms. For example, if the authority is courteous, it adds to the standing of the party in question. Research shows a strong correlation between courtesy and procedural justice.^48^

The relational model provides a fascinating view on people’s estimation of authority: it turns out to be complex and not simply determined by how much the decision maker favours them in the decision(s). However, at the same time, the model offers a somewhat *bleak* picture of how people assess the quality of the decision. To put the point bluntly: recognition by the decision maker of a person’s standing turns out to be a more important factor than whether the decision itself is good. There is also a considerable danger lurking in the shadows. By inducing the belief that the parties in question have been truly heard, the decision maker may enhance its legitimacy while at the same time deciding the matter in a questionable manner. Tyler and Lind are in fact well aware of possible manipulation and provide the reader with a caution:

For followers, the principal message of our work is a caution. The relational model argues that authority turns on factors that are subjective and stylistic in nature. This raises a problem of what is often termed *false consciousness* or *hollow justice*. Followers have to be on guard to protect against unscrupulous leaders who offer a false image of concern for their welfare.^49^

This caution—which is not taken up by either Erez or Wemmers—is very much in line with the argument we developed in the previous section. The fact that people value X in terms of procedural fairness does not necessarily mean—in light of the fact–value distinction—that X adds to the fairness of proceedings, because X may be an example of hollow justice. Since perceptions of procedural justice are to a large extent determined by the standing accorded to the person in question, empirical ‘proof’ of perceived fairness does not guarantee that the decision was of good quality. This, again, is not to say that people are constantly mistaken in identifying procedures as (un)fair, but it does mean—if the ‘is–ought’ gap is to be avoided—that the development of a critical normative conception of ‘procedural justice’ is required. Another reason why a normative conception is necessary is the worry that VIS may actually be an example of hollow justice. Erez herself, for example, complains that judges tend to ignore VIS to such an extent that it amounts to no more than ‘placebo justice’.^50^ However, if that is how we should assess VIS, invoking the argument of procedural justice seems questionable.

Before turning to the development of a normative conception of procedural justice, there is another issue which deserves some attention. Tyler and Lind’s analysis indicates a collapse of the procedural justice argument into the argument of therapeutic jurisprudence. At the start of the section, we suggested that procedural justice takes priority over therapeutic jurisprudence, but the arguments now turn out to be two sides of the same coin.^51^ After all, there is a connection not only between perceived procedural justice and social standing, but also between standing and recovery. By reporting that VIS adds to the fairness of the proceedings, the victim is effectively making a statement about the status accorded to her by the process, which, in turn, facilitates her recovery. Also, it emerges that the argument of procedural justice cannot bridge the gap between the legal and psychological domain because, as developed by Erez and Wemmers, it remains firmly rooted in the psychological domain. The question of how VIS relates to a normative conception of procedural justice and to the corresponding values of the legal system therefore becomes all the more pertinent.

A famous normative and critical analysis of procedural justice is provided by John Rawls. In *A Theory of Justice*, he makes a distinction between *perfect* and *imperfect* procedural justice.^52^ According to Rawls, a procedure is perfectly fair if (i) an independent standard is available to determine what should count as the right result and (ii) a procedure can be developed which is capable of generating this result. Rawls illustrates this with the example of dividing a cake. Suppose that the parties in question should all receive an equal slice. In that case, from a procedural justice point of view, it would be perfectly fair if the person cutting the cake—assuming that she is able to cut equal slices—would get to choose last. The criminal trial, on the other hand, is an example of imperfect justice. An independent standard is available—a defendant should only be convicted if in fact guilty—but it is impossible to devise a fail-safe procedure that guarantees that this standard is met. Typically, a trial is a fallible enterprise where sometimes defendants who should have been acquitted are convicted, and *vice versa*. It thus emerges that Rawls’s interpretation of procedural justice diverges considerably from Tyler and Lind’s model. In contrast to their model, Rawls understands procedural justice entirely in instrumental terms: a procedure is fair if it generates the right result or, at least, is better at generating the right result than any other procedure.

Jürgen Habermas presents a slightly different take on procedural justice in his comprehensive work *Between Facts and Norms.*^53^ According to Habermas, procedural law should enable an impartial and independent judge to form an opinion which can be regarded as the reasonable conclusion of a free exchange of arguments within the regulated sphere of the procedure. Symmetry should be observed in relation to the input of the parties (plaintiff and defendant in civil law; prosecutor and accused in criminal law). The restrictions that are in play within the procedure may not, in Habermas’s view, upset or influence the soundness of the reasoning in the process of argumentation. Only by being based on a fair and balanced procedure in which the best argument counts can the judge’s verdict on both the facts and the applicable law be considered legitimate. A free exchange of arguments and the possibility of rebutting each other’s claims are therefore central to Habermas’s conception of procedural justice. While Habermas’s analysis connects better than Rawls’s with Tyler and Lind’s relational model, some differences can be discerned. A striking difference is that in Habermas’s analysis it is impossible to detach outcome (verdict) from procedure. The two are not only factually, but also normatively connected: the legitimacy of the verdict is dependent on the fairness of the procedure, but also the other way around: without a verdict, the procedure would be deprived of its point. So, if we follow Habermas’s analysis, not just *any* statement made during the judicial process can be said to contribute to procedural justice, but only contributions which (i) are relevant to the verdict of the judge and (ii) do not disturb the equal balance between the parties within the procedure itself. Being presented with the opportunity of making such a contribution can be regarded as intrinsically valuable, because it offers the persons in question the possibility of presenting and recognising themselves and others as responsible legal subjects.^54^

When we consider VIS in the light of these critical normative theories of procedural justice, a different picture emerges than that which Erez and Wemmers present. VIS does not so much enhance procedural justice, but rather puts procedural justice under pressure. We illustrate this by discussing two different formats of VIS.^55^

In Canada, victims are able to make a statement in the sentencing phase. This right is limited, however. Victims may describe how the crime has affected their lives, but they are not allowed to make any allegations or include a recommendation regarding the sentence.^56^ Judges, in turn, are to consider VIS in order to develop a better understanding of the harm involved, but it may not affect their sentencing. For example, it should not result in harsher punishment.^57^ This format does not connect too well with either Rawls’s or Habermas’s analysis of procedural justice. In Rawls’s analysis, VIS would be fair from a procedural point of view if it were to have a positive influence on sentencing. But since VIS in this format is not meant to have any effect, it cannot be understood in terms of procedural justice. In Habermas’s analysis, VIS adds to procedural justice if it would balance out the discussion game played in court in which every argument is open to contestation. But it is hard to see how VIS could introduce a greater balance as it is a one-sided statement which is not meant to be contested in court. In defence of Erez and Wemmers, it could be argued that VIS at least provides voice and process control to the victim. But can this argument withstand scrutiny? Within the meaning Robert Folger originally applied to the notion ‘voice’, it would be applicable only if the person expressing her opinion somehow actively *participates* in the decision-making process.^58^ Since the legal relevance of VIS is limited, it seems questionable whether delivering a VIS can be regarded as ‘participating in decision making’. John Thibaut and Laurens Walker have introduced the notion of process control: a person has more or less process control if she can influence—to a greater or lesser extent—the information on which the authority decides the issue.^59^ If we apply this way of understanding the notion to the Canadian context, it emerges that VIS provides little process control to the victim, unlike, for example, a witness who knows that her testimony will be taken into account as part of the decision-making process.

A less restrictive format of VIS has been adopted in the United States. Just as in Canada, victims can make an impact statement during the sentencing phase, but in most states victims are met with very few restrictions on what they are allowed to say and the means to deliver their message.^60^ In murder cases, next of kin may present evidence about the victim’s character and standing in her community and explain in detail how the murder has affected them.^61^ In some states, they are also allowed to propose a sentence, and the judge and jury can take this proposal into account.^62^ This permissive approach finds its basis in the US Supreme Court’s controversial ruling in *Payne v Tennessee*, in which the Court held that VIS can withstand constitutional scrutiny in capital cases.^63^ According to the Supreme Court, VIS offers a glimpse of the life of the victim, and as such offers relevant evidence as to the harm that was caused. This is not to say that there are no restrictions in play. For example, the content of the VIS may not be so unduly prejudicial that it renders the trial fundamentally unfair. This format of VIS indeed offers some process control because it may influence the sentence. Does this contribute to the fairness of the legal process? A common criticism of this way of organising sentencing is that it could result in *disproportionate* punishment.^64^ If the victim is apt in conveying the harm that was caused, the offender may receive a stiffer punishment compared to other offenders. A related *normative* concern is that VIS may interfere with the symbolic dimension of sentencing. In our estimation, there is merit to the idea that the purpose of punishment is not primarily to requite the private harm of a particular victim, but rather to publicly express how the offence is assessed from a general point of view, as exemplified by the generality of law itself.^65^ This public, symbolic aspect may fade into the background if VIS takes centre stage. While the discussion about this format of VIS tends to concentrate on the empirical question of how it could affect the length of the sentence, in our estimation this normative concern is just as important. All of this suggests that it is debatable whether this format of VIS fits well with procedural justice.

These two examples indicate that it can be difficult to reconcile VIS and procedural justice. If VIS has no or very little influence on the sentencing, it cannot substantially contribute to the fairness of the outcome of the legal procedure. If it does exert an influence, it can be unfair if it concerns a statement which cannot be properly *included*—contested, assessed or rebutted—in the course of a legal procedure in which participants are treated equally and the best argument should prevail. Erez, however, does not seem interested in this fairness concern: in her view, the scope of victim participation should be extended considerably. Empowerment of the victim includes, she maintains, that she should be heard in *all* stages of the legal process.^66^ However, such an expansion of VIS would be problematic from a procedural justice point of view as it violates one of the central principles warranting the fairness of the trial: the presumption of innocence. Suppose, for example, that the victim can make a statement during the trial itself and addresses the defendant as the perpetrator. The problem with such a statement is that it anticipates a guilty verdict. The position of the judge is also compromised as she is required to take up an ambiguous stance. On the one hand, she is expected to listen attentively to what the victim has to say and show respect to her truth claims as an—already—acknowledged crime victim. On the other hand, however, she has to safeguard her impartiality by making clear to the defendant that she is still presumed innocent and that the legal truth is yet to be established in the course of a fair procedure.^67^ Erez’s recommendation thus does not match well with compelling normative ideas of procedural justice, as developed by Rawls and, in particular, Habermas. In these theories the legitimacy of the outcome—the legal truth as laid out in the verdict—and the neutrality and fairness of the procedure—in which the best argument should count on the basis of a reasoned and well-regulated contestation in a context of equality of arms between parties—are inextricably connected.^68^

In the previous section, we argued that a number of authors failed to properly consider the humiliating character of the ordered apology due to their empirical orientation. We can now draw a similar conclusion concerning the discussion about VIS. Erez and Wemmers turn out to be so concerned with victims’ well-being and the satisfaction of their needs that they fail to consider the possibility of VIS *not* enhancing the fairness of legal proceedings. In their view, procedural justice means empowering victims by providing them with standing. However, what is fair cannot be determined by considering the factual needs and wishes of only one of the parties involved.^69^ Rather, a sound conception of procedural justice includes an equal concern and respect for all parties and for the general point or collective goal of the procedure as a whole. Together, the parties bear a shared responsibility of providing the judge with the opportunity of reaching a valid and non-partisan decision—a decision which can be regarded as the result of a free, fair and balanced exchange of arguments. All parties are therefore required to answer for what they contribute to the proceedings. In the formats discussed above, VIS eithers fails or leaves a lot of room for doubt. Rather than enhancing the fairness of proceedings, a tension between the empirical underpinnings of VIS and the normative foundation of procedural justice can be discerned. Due to their empirical orientation, Erez, Wemmers and others fail to appreciate this tension and even reach a diametrically opposed conclusion. By equating procedural justice with victim perceptions of procedural justice, they overlook the pre-eminently normative character of the concept and its overarching, general and impartial goals.

## 4. Conclusion

Wherever we look in the society of the present: what is expected more and more is not the general, but the particular. The hopes, the interest and the efforts of institutions and individuals are not attached to what is standardised and regulated, but to the unique, the singular.^70^

With the above-mentioned examples, we have aimed to show that empirical-legal research with regard to victims can suffer from a blind spot, as well as from bias. The strong focus on the measurable needs of victims can lead to a predominantly instrumental approach of law and the legal system. Within this approach, the legal system has to be increasingly tailored to the wishes and needs of particular victims, despite the awareness that the singular suffering of victims can never be fully remedied by any legal procedure. Within this victim-oriented approach, little attention is paid to the general normative principles and overarching goals of the legal system within liberal democracies, in which legitimate and impartial decisions on the one hand and legal procedures based on the equal treatment of persons as free and responsible legal subjects on the other hand are inextricably connected. These overarching aims connect such a fundamental human right as the prohibition of inhumane or degrading treatment or punishment with procedural legal principles such as the right to be heard (*audi et alteram partem*), the principle of equality of arms and the presumption of innocence.^71^ Moreover, due to the fixation on victims and their psychological needs within a substantial part of empirical-legal research, the view of human beings as free and responsible persons—answerable for their truth claims and defaults—as the cornerstone of modern legal systems risks becoming undervalued.^72^

This development deserves critical reflection, not only because legal frameworks which would *unilaterally* serve the needs and desires of victim groups could lead to infringements on fundamental freedoms, partisan and biased trials and institutionalised cruelty, but also because a well-functioning and robust rule of law system requires constant normative maintenance. Empirical research *can* enrich legal scholarship, provided that empirical results are not too easily or too quickly translated into proposals for legal reform, but rather become part of a hermeneutical discussion about norms which is specific to legal scholarship. In this regard, it is important to note that attention should be paid not only to the current state of positive law and legal doctrine, but also to the underlying principles and values of the legal system as a whole or of the legal area involved, and to the corresponding view of the human person. In light of the two examples of empirical-legal research discussed in this article, we observe that the gap between the factual and singular on the one hand and the normative and general on the other hand, between empirical data and legal conclusions, between behavioural sciences and hermeneutical legal scholarship and jurisprudence, or simply between ‘is’ and ‘ought’, is a notoriously difficult one to bridge, an endeavour that may only be brought about in a defensible way by uniting forces in a truly multidisciplinary way, by coherently bringing together sufficient in-depth contributions from various disciplines.^73^

Finally: how and to what extent the needs of victims should be addressed within a humane legal order remains the subject of a lively debate that partly extends beyond the legal domain. Is the courtroom the most appropriate place for victims to receive recognition and support for the processing of their experiences? Should victims not have legal responsibilities when allowed to make statements in court on all aspects of the truth finding process and the possible verdict? And, *last but not least*: how do we prevent the political and empirical-legal desire to support and empower victims from transforming the legal procedure in such a way that it could start to produce its own victims? The fundamental discussion about these questions is of great importance and, we believe, not even nearly finished.

## Footnotes

1 Stephen Schafer makes such a claim in his classical work *The Victim and His Criminal* (Random House 1968) ch 1. Interestingly, the focus of the pioneers of victimology was not only on providing victims with better access to the legal arena, but also on establishing how victims contribute to their own victimisation.

2 The World Society of Victimology formulates its commitment as follows: ‘The membership of the World Society of Victimology consists of a wide variety of individuals—from students to academics, from interested individuals to government officials—dedicated to improving the plight of victims of crime and the abuse of power around the globe.’ <www.worldsocietyofvictimology.org/welcome/who-we-are/> accessed 10 June 20.

3 See eg Tamara Relis, ‘“It’s Not about the Money!”: A Theory on Misconceptions of Plaintiff’s Litigation Aims’ (2007) 68 U Pitt L Rev 701.

4 Gijs van Dijck, ‘The Ordered Apology’ (2017) 37 OJLS 562.

5 Andrea Zwart-Hink, Arno Akkermans and Kiliaan van Wees, ‘Compelled Apologies as a Legal Remedy: Some Thoughts from a Civil Law Jurisdiction’ (2014) 38 UWAL Rev 100; Aaron Lazare, *On Apology* (OUP 2004); Brent T White, ‘Say You’re Sorry: Court-Ordered Apologies as a Civil Rights Remedy’ (2006) 91 Cornell L Rev 1261.

6 Nicholas Tavuchis considers voluntariness a necessary condition in his influential analysis of apology. He states that ‘To apologize is to declare *voluntarily* that one has no excuse, defense, justification, or explanation for an action that has insulted, failed, injured, or wronged another …’ Nicholas Tavuchis, *Mea Culpa: A Sociology of Apology and Reconciliation* (Stanford UP 1991) 17 (emphasis added). See also Mano Daniel and Jim Gough, ‘Forgiveness, Finitude, Apology and Acknowledgment’ (2017) 1 CSSPE/SCEEA 180, 192. They hold that an apology can only be genuine if ‘It is sincere, authentic or self-authored by the person giving the apology. The mother apologizing for the actions of her grown son, without the express permission of the son, may be an example of an insincere apology by proxy.’

7 Van Dijck (n 4) 570.

8 This is different with children. In fact, it is very common for a parent or a teacher to ask the child to apologise. We return to this difference between adults and children later on in the text.

9 Van Dijk and also Zwart-Hink, Akkermans and Van Wees call upon research performed by—among others—Jane Risen and Thomas Gilovich to substantiate this claim. Risen and Gilovich’s studies demonstrate that targets (as opposed to observers) do not clearly differentiate between spontaneous and compelled apologies. See Jane L Risen and Thomas Gilovich, ‘Target and Observer Differences in the Acceptance of Questionable Apologies’ (2007) 92 Journal of Personality and Social Psychology 418.

10 Van Dijck (n 4) 573–4. We do not discuss the possible beneficial consequences to society in this article.

11 ibid 580–5. See also Zwart-Hink, Akkermans and Van Wees (n 5) 109–19.

12 Moore argues in this way when he discusses the retributive sentiment. He states that ‘The familiar argument that in the design of punishment institutions we ought to capitulate to the felt resentments of most citizens has been answered many times before. If the popular judgment that results count is wrong, then *we ought to educate citizens to the right view, not reinforce them in their error*.’ Michael S Moore, *Placing Blame: A Theory of the Criminal Law* (OUP 1997) 207 (emphasis added).

13 Van Dijck (n 4) 574.

14 Lazare (n 5) 225–6 (emphasis added).

15 Major Ted Conway recounts: ‘General Patton started to give what we knew was to be his apology. But he never got past his first word, which was “Men!” At that point, the whole regiment erupted. It sounded like a football game in which a touchdown has been scored … Then he had the bugler sound “attention” again, but nothing happened. Just all these cheers. So, finally General Patton was standing there and he was shaking his head and you could see big tears streaming down his face and he said, or words to this effect, “The hell with it,” and he walked off the platform. At this point the bugler sounded “attention” and everybody grabbed the nearest available steel helmet, put it on, being sure to button the chin strap (which was a favorite Patton quirk) and as he stepped into his command car and again went down the side of the regiment, dust swirling, everybody stood at attention and saluted to the right and General Patton stood up in his command car and saluted, crying … He was our hero. *We were on his side. We knew the problem. We knew what he had done and why he had done it*.’ DA Lande, *I Was With Patton: First-Person Accounts of WWII in George S Patton’s Command* (MBI Publishing Company 2002) 90–1 (emphasis added).

16 Art 1 of the Charter of Fundamental Rights of the European Union states that ‘Human dignity is inviolable. It must be respected and protected.’

17 See art 3 ECHR. According to the European Court of Human Rights, a custodial sentence does not violate this article as long as the physical and moral integrity of the detainee is respected. He may not be required to do or say things which violate his core values. See Jim Murdoch and Vaclav Jiricka, *Combating Ill-Treatment in Prison: A Handbook for Prison Staff with Focus on the Prevention of Ill-Treatment in Prison* (Council of Europe 2016); see also Alison Liebling, ‘Moral Performance, Inhuman and Degrading Treatment and Prison Pain’ (2011) Punishment & Society 530.

18 See Zwart-Hink, Akkermans and Van Wees (n 5) 122.

19 Van Dijck (n 4) 565.

20 See below for a discussion.

21 In contrast to Van Dijck, White—albeit reluctantly—limits his plea in favour of the ordered apology to *public* actors. See White (n 5) 1298. While it is beyond the scope of this article to discuss the relevance of the distinction between public and private actors in this area, our view is that ordering a public actor to apologise is also beset by problems. The Patton apology is a case in point. In the same vein, see Martha Minow, *Between Vengeance and Forgiveness: Facing History after Genocide and Mass Violence* (Beacon Press 1998) 112: ‘the problems with apology [from public actors] include insincerity, an absence of clear commitment to change, and incomplete acknowledgment of wrongdoing … Perhaps most troubling are apologies that are purely symbolic, and carry no concrete shifts in resources or practices to alter the current or future lives of survivors of atrocities.’

22 White (n 5) 1274 (emphasis added).

23 ibid 1275 and 1286 (emphasis added).

24 Immanuel Kant, *The Metaphysics of Morals* (Mary J Gregor ed, CUP 1996) 105.

25 Smith criticises Kant as follows: ‘Humiliation when ordered to kiss the hand of an inferior holds quite different content. I am humiliated because I am paraded as a subject of the court. Rather than reintegrating me into the moral community, such degradation seems more likely to stoke resentment and alienation. Instead of the state helping to understand my blameworthiness and cultivating the appropriate constructive emotions and actions that should follow, it drags me down a path of humiliation through the spectacle of my punishment.’ Nick Smith, *Justice through Apologies: Remorse, Reform, and Punishment* (CUP 2014) 61.

26 Linda Radzik, *Making Amends: Atonement in Morality, Law and Politics* (OUP 2009) 18.

27 For an interesting exposition on rehearsal, script and choreography of compelled public confessions in criminal cases in China, see Magnus Fiskesjö, ‘The Return of the Show Trial: China’s Televised “Confessions”’ (2017) 15 Asia-Pacific Journal: Japan Focus 1.

28 Avishai Margalit, *The Decent Society* (HUP 1996) 1.

29 ibid 108–12.

30 See eg Jeremy Waldron, ‘Cruel, Inhuman, and Degrading Treatment: The Words Themselves’ (2008) NYU School of Law, Public Law Research Paper 1, 38: ‘an adult has achieved full human status and is capable of standing upright on his or her own account, in a way that (say) an infant is not. So it is degrading to treat an adult human as though he or she were an infant or in ways appropriate to treating an infant.’

31 See Dienke Hondius, *Blackness in Western Europe: Racial Patterns of Paternalism and Exclusion* (Trans Pub 2014).

32 Reidy bases her definition of degrading treatment on the case law of the European Court of Human Rights: ‘Degrading treatment is that which is said to arouse in its victims feelings of fear, anguish and inferiority, capable of humiliating and debasing them. This has also been described as involving treatment such would lead to breaking down the physical or moral resistance of the victim … *or as driving the victim to act against his will or conscience*.’ Aisling Reidy, *The Prohibition of Torture: A Guide to the Implementation of Article 3 of the European Convention on Human Rights* (Council of Europe 2002) 16 (emphasis added).

33 Van Dijck (n 4) 585. White also stresses the appropriateness of courts ordering defendants to apologise because parents do so too with their children. White (n 5) 1284.

34 Margalit (n 28) 149.

35 Dwarf-tossing may be an example of humiliating behaviour—the person is used as if he is an object—that is not always recognised as such by the people involved.

36 See Sanne Taekema, ‘Theoretical and Normative Frameworks for Legal Research: Putting Theory into Practice’ [2018] Law and Method 1; David Dyzenhaus, ‘Dworkin and Unjust Law’ in Wil Waluchow and Stefan Sciaraffa (eds), *The Legacy of Ronald Dworkin* (OUP 2016).

37 Bandes’s critique of VIS in capital punishment cases is especially influential. See Susan Bandes, ‘Empathy, Narrative, and Victim Impact Statements’ (1996) 63 U Chi L Rev 361; Susan A Bandes, ‘Victims, Closure, and the Sociology of Emotion’ (2009) 72 LCP 1. A critical discussion of VIS and victim talk in general can be found in Martha Minow, ‘Surviving Victim Talk’ (1993) 40 UCLA L Rev 1411.

38 eg Edna Erez and Pamela Tontodonato, ‘Victim Participation in Sentencing and Satisfaction with Justice’ (1992) 9 JQ 393.

39 Edna Erez, ‘Who’s Afraid of the Big Bad Victim? Victim Impact Statements as Victim Empowerment and Enhancement of Justice’ [1999] Crim LR 545, 551; Jo-Anne Wemmers and Marie-Marthe Cousineau, ‘Victim Needs and Conjugal Violence: Do Victims Want Decision-Making Power?’ (2005) 22 Conflict Resolution Quarterly 493, 495.

40 Wexler defines therapeutic jurisprudence as the study of ‘law’s impact on emotional life and psychological well-being’. See David B Wexler, ‘Therapeutic Jurisprudence and its Application to Criminal Justice Research and Development’ (2010) 7 Irish Probation Journal 94, 95.

41 See Erez, ‘Who’s Afraid of the Big Bad Victim? (n 39) 552–3; Jo-Anne Wemmers, ‘Victim Participation and Therapeutic Jurisprudence’ (2008) 3 Victims & Offenders 165, 186–7. See also Antony Pemberton and Sandra Reynaers, ‘The Controversial Nature of Victim Participation: Therapeutic Benefits in Victim Impact Statements’ in Edna Erez, Michael Kilchling and Jo-Anne Wemmers (eds), *Therapeutic Jurisprudence and Victim Participation in Justice* (Carolina Academic Press 2011).

42 Erez states that ‘Research conducted in the United States and Australia on victims of various crimes, where a VIS is relevant (ie a personal harm of loss was suffered by a specific victim), suggests that victims are interested in having a voice … The English victim statements pilot project confirms this finding … Proceedings which provide victims with a voice or “process control” enhance their satisfaction with justice and sense of fair treatment.’ Erez, ‘Who’s Afraid of the Big Bad Victim?’ (n 39) 551.

43 Jo-Anne Wemmers, *Victims in the Criminal Justice System* (Kugler Publications 1996) 136–43.

44 Tom R Tyler and E Allan Lind, ‘A Relational Model of Authority in Groups’ (1992) 25 Advances in Experimental Social Psychology 115. Tyler and Lind, in turn, build on the work of Thibaut and Walker. Interestingly, Thibaut and Walker do not focus exclusively on perceptions of procedural justice in their analysis. See John Thibaut and Laurens Walker, *Procedural Justice: A Psychological Analysis* (Lawrence Erlbaum Associates 1975).

45 Tyler and Lind (n 44) 117–39.

46 The results of this experiment were first published in E Allan Lind, Ruth Kanfer and P Christopher Earley, ‘Voice, Control and Procedural Justice: Instrumental and Noninstrumental Concerns in Fairness Judgments’ (1990) 59 Journal of Personality and Social Psychology 952.

47 Tyler and Lind state: ‘It appears that the opportunity to express one’s opinions and arguments, the chance to tell one’s own side of the story, is a potent factor in enhancing the experience of procedural justice, even when the opportunity for expression really accomplishes nothing outside the procedural relationship. Indeed, it is now clear that the opportunity to state one’s case may be valued as much or more for its own sake as for its instrumental influence on decisions.’ Tyler and Lind (n 44) 149. However, this effect does not occur when it is very clear that the decision maker is unfair.

48 Tyler and Lind (n 44) 139–43.

49 ibid 162, original emphasis (references omitted).

50 Erez states that ‘There is also no evidence that including victims in the criminal justice process results in punitiveness or that requests by victims for harsh sanctions influence the sentences. The characterization of victims’ rights for input as “placebo justice” is more consistent with reality.’ Edna Erez, ‘Victim Participation in Sentencing: Rhetoric and Reality’ (1999) 18 JCJ 19, 29.

51 This collapse is—perhaps inadvertently—acknowledged by Wemmers in a joint publication with Cyr. See Jo-Anne Wemmers and Katie Cyr, ‘Can Mediation Be Therapeutic for Crime Victims? An Evaluation of Victims’ Experiences in Mediation with Young Offenders’ (2005) 47 Canadian Journal of Criminology and Crimin Justice 527, 540.

52 John Rawls, *A Theory of Justice* (rev edn, OUP 1999) 74–5.

53 Jürgen Habermas, *Between Facts and Norms: Contributions to a Discourse Theory of Law and Democracy* (William Rehg tr, MIT Press 1996) 234–6.

54 Ricoeur stresses the significance of having access to juridical institutions as a condition for personal development. However, it is only in relation to others—for example, by being required to take up different roles or having to negotiate—that this development can take place. See Paul Ricoeur, *The Just* (UCP 2000) 1–10.

55 In the examples, we do not consider the restitution the victim may ask for the losses incurred. Also, we do not describe the formats in more detail than is required for our general discussion.

56 For a more detailed description of Canadian criminal justice, see Wemmers, ‘Victim Participation and Therapeutic Jurisprudence’ (n 41) 168–70.

57 Justice Bouck, for example, states in *R v Labbe* (2001) 160 BCAC 61 (CA) that ‘victim impact statements are essential for two purposes. First, so the court is more aware of the harm done by the offender to the victim so that the sentencing judge has a better understanding of the offence’s gravity. Second, to assure victims that the sentencing process includes them by ensuring they are not irrelevant and forgotten … Furthermore, it is not clear whether a victim’s impact statements should have any effect on a sentence for manslaughter. This is because the law should recognize the equal value of every life that is taken whether the victim’s survivors grieve or not.’

58 Robert Folger, ‘Distributive and Procedural Justice: Combined Impact of “Voice” and Improvement on Experienced Inequity’ (1977) 35 Journal of Personality and Social Psychology 108, 109. See also Albert O Hirschman, *Exit, Voice, and Loyalty: Responses to Decline in Firms, Organizations, and States* (Harvard UP 1970).

59 Thibaut and Walker, for example, describe that disputants in an adversarial system have more process control than in an inquisitorial system. See Thibaut and Walker (n 44) 22–7. See also Pauline Holden and others, ‘Preference for Modes of Dispute Resolution as a Function of Process and Decision Control’ (1978) 14 Journal of Experimental Social Psychology 13.

60 Bandes mentions a case ‘involving victim impact testimony in the form of an emotionally powerful film about the life of the victim, including music, a voiceover by the victim's mother, and a concluding shot of wild horses running free.’ Bandes, ‘Victims, Closure, and the Sociology of Emotion’ (n 37) 22–3.

61 See John H Blume, ‘Ten Years of Payne: Victim Impact Evidence in Capital Cases’ (2003) 88 Cornell L Rev 257, 269–70. See also Angela P Harris, ‘The Jurisprudence of Victimhood’ [1991] Sup Ct Rev 77, 95–6.

62 Blume (n 61) 276.

63
*Payne v Tennessee* 501 US 808 (1991). This ruling reversed the Supreme Court’s earlier decision in *Booth v Maryland* in which the Supreme Court argued that VIS could ‘divert the jury’s attention away from the defendant’s background and record, and the circumstances of the crime’. *Booth v Maryland* 482 US 496 (1987).

64 There is a debate about whether this criticism is correct. Some studies report no effect: see Trina M Gordon and Stanley L Brodsky, ‘The Influence of Victim Impact Statements on Sentencing in Capital Cases’ (2007) 7 Journal of Forensic Psychology Practice 45. Other studies reveal that the presence of a VIS results in lengthier sentences: Amy Wevodau and others, ‘The Role of Emotion and Cognition in Juror Perceptions of Victim Impact Statements’ (2014) 27 Social Justice Research 45.

65 The distinction between public and private harm is central to Duff’s thinking about criminal law. See eg RA Duff, ‘Perversions and Subversions of Criminal Law’ in RA Duff and others (eds), *The Boundaries of the Criminal Law* (OUP 2010) 90–2.

66 Erez states that ‘The victim has to be brought back into the process in all the stages that impact the case outcome, whether charging, dismissal of charges, plea bargaining, or sentencing … The empowering of victims to have a say in all important decision making points, as some European countries allow, is necessary for victims’ recognition and restoration. Otherwise, as the experience with other victims’ rights has shown, the need to be heard may become a continuous source of frustration for victims who feel that their input is important or believe they should at least be given an opportunity to influence the case outcome and then discover that no one is interested in hearing what they have to say, let alone willing to consider it.’ Erez, ‘Victim Participation in Sentencing’ (n 50) 29 (references omitted).

67 The ECHR considers it a violation of the presumption of innocence if ‘there is some reasoning suggesting that the court regards the accused as guilty’. See *Minelli v Switzerland* (1983) Series A no 62. In Erez’s proposal, it would not be easy for the judge to heed this command.

68 Shapland connects strong opposition against VIS with banning emotion from the court. However, our opposition should not be interpreted in this way. Our concern is with fairness. See Joanna Shapland, ‘Victims and Criminal Justice in Europe’ in Shlomo Giora Shoham, Paul Knepper and Martin Knett (eds), *International Handbook of Victimology* (CRC Press 2010) 365.

69 Ashworth aptly states: ‘However, the touchstone of these claims should not be the wishes of victims so much as the nature and goals of the criminal process as a legal and social institution.’ Andrew Ashworth, ‘Victim Impact Statements and Sentencing’ [1993] Crim LR 499.

70 Andreas Reckwitz, *Die Gesellschaft der Singularitäten* (Suhrkamp Verlag 2017) 7: ‘Wohin wir auch schauen in der Gesellschaft der Gegenwart: Was immer mehr erwartet wird, ist nicht das Allgemeine, sondern das Besondere. Nicht an das Standardisierte und Regulierte heften sich die Hoffnungen, das Interesse und die Anstrengungen von Institutionen und Individuen, sondern an das Einzigartige, das Singuläre’ (our translation).

71 See Tom Bingham, *The Rule of Law* (Allen Lane 2010) 90–109.

72 See Lon Fuller, *The Morality of Law* (rev edn, Yale UP 1969) 162; Sean Coyle, *Modern Jurisprudence: A Philosophical Guide* (Hart Publishing 2014) 206–7.

73 For an example in which such an integrated approach is virtually absent, see Martin Partington, ‘Empirical Legal Research and Policy-Making’ in Peter Cane and Herbert Kritzer (eds), *The Oxford Handbook of Empirical Legal Research* (OUP 2010) 1003. Interesting reflections on how to bridge the divide between empirical facts and legal norms are offered by David Nelken, ‘Can Law Learn from Social Science?’ (2001) 35 Israel Law Review 205; Edward Rubin, ‘Law and the Methodology of Law’ (1997) 3 Wis L Rev 521; Ivo Giesen, ‘The Use and Incorporation of Extralegal Insights in Legal Reasoning’ (2015) 11 Utrecht Law Review 1; Bart van Klink, Sanne Taekema and Wouter de Been, ‘Introduction’ in Bart van Klink, Sanne Taekema and Wouter de Been (eds), *Facts and Norms in Law: Interdisciplinary Reflections on Legal Method* (Edward Elgar 2016) 3.

